# Factors associated with survival and hospital discharge amongst critically ill patients undergoing prolonged mechanical ventilation in the North of England Critical Care Network

**DOI:** 10.1186/cc14335

**Published:** 2015-03-16

**Authors:** L O'Connor, I Gonzalez, L Garcia

**Affiliations:** 1 Sunderland Royal Hospital, Sunderland, UK; 2 James Cook University Hospital, Middleborough, UK

## Introduction

The combination of a global demographic shift and increased survival following critical illness has led to an increasing number of patients requiring prolonged mechanical ventilation (PMV) and longer critical care stay. This is a prospective observational study evaluating the characteristics and speciality-based outcome of critically ill patients undergoing prolonged mechanical ventilation in the North of England Critical Care Network (NoECCN).

## Methods

A weekly survey was conducted over a 1-year period screening patients older than 16 years of age requiring PMV in all 18 adult critical care units within the NoECCN. Patient data collected included patient demographics, admission diagnosis and speciality, hospital length of stay (LOS) pre and post critical care admission, severity of illness scores, critical care LOS and status at hospital discharge.

## Results

During the study period 134 patients met the criteria for PMV representing 1% of annual admissions and 6.9% NoECCN bed-days. The majority of patients receiving PMV were medical (50.7%), followed by emergency surgery (20.1%), elective surgery (16.4%) and specialist services such as spinal cord injury (8.2%) and cardiothoracic transplant (4.5%). The commonest admission diagnosis in the medical population was pulmonary infection followed by acute neurological disorders, while 89.4% of surgical patients were admitted to critical care during the perioperative period. At the end of the study period the highest hospital mortality was observed in the nonspecialist surgical population (26.5%). In contrast, the medical population had one of the lowest hospital mortality rates (11.8%), lower than predicted using the intensive care national audit research network illness severity score. Comparable rates of hospital discharge were found in both medical (85%) and nonspecialist surgical patients (88.9%).

## Conclusion

The results of this study highlight an expanding proportion of NoECCN critical care bed-days occupied by stable patients undergoing PMV. In keeping with published UK data, elevated hospital mortality was observed in the nonspecialist surgical subpopulation. Although the literature suggests the medical cohort of patients has poorer prognosis, within our region all were liberated from mechanical ventilation and over 80% were discharged from hospital.
